# GSHFA-HCP: a novel intelligent high-performance clustering protocol for agricultural IoT in fragrant pear production monitoring

**DOI:** 10.1038/s41598-024-66631-8

**Published:** 2024-07-20

**Authors:** Peng Zhou, Wei Chen, Jing Wang, Huan Wang, Yunfeng Zhang, Bingyu Cao, Shan Sun, Lina He

**Affiliations:** 1 School of Information Science and Engineering, Xinjiang College of Science & Technology, Korla, 841000 Xinjiang China; 2 School of Computer, North China Institute of Aerospace Engineering, Langfang, 065000 Hebei China

**Keywords:** Agricultural Internet of Things, Wireless sensor network, Clustering routing, Firefly algorithm, Energy consumption, Network lifetime, Computational science, Computer science, Information technology, Software

## Abstract

The agriculture Internet of Things (IoT) has been widely applied in assisting pear farmers with pest and disease prediction, as well as precise crop management, by providing real-time monitoring and alerting capabilities. To enhance the effectiveness of agriculture IoT monitoring applications, clustering protocols are utilized in the data transmission of agricultural wireless sensor networks (AWSNs). However, the selection of cluster heads is a NP-hard problem, which cannot be solved effectively by conventional algorithms. Based on this, This paper proposes a novel AWSNs clustering model that comprehensively considers multiple factors, including node energy, node degree, average distance and delay. Furthermore, a novel high-performance cluster protocol based on Gaussian mutation and sine cosine firefly algorithm (GSHFA-HCP) is proposed to meet the practical requirements of different scenarios. The innovative Gaussian mutation strategy and sine–cosine hybrid strategy are introduced to optimize the clustering scheme effectively. Additionally, an efficient inter-cluster data transmission mechanism is designed based on distance between nodes, residual energy, and load. The experimental results show that compared with other four popular schemes, the proposed GSHFA-HCP protocol has significant performance improvement in reducing network energy consumption, extending network life and reducing transmission delay. In comparison with other protocols, GSHFA-HCP achieves optimization rates of 63.69%, 17.2%, 19.56%, and 35.78% for network lifespan, throughput, transmission delay, and packet loss rate, respectively.

## Introduction

The Internet of Things (IoT) constitutes a network comprised of various physical devices embedded with sensors, software, and connectivity, enabling the collection and exchange of data. With the continual advancement of IoT technology, it has progressively permeated various industries and domains, including manufacturing, healthcare, transportation, and agriculture. Concurrently, the emergence of 5G and 6G technologies has ushered in a new era of connectivity^1^, presenting unprecedented opportunities for the development of IoT, particularly within the agricultural domain. Leveraging the capabilities of supporting a multitude of connected devices, ultra-low latency, and higher data rates, these next-generation networks have the potential to revolutionize agricultural practices through real-time monitoring, precision agriculture, and intelligent decision support systems.

Agriculture IoT is the concept of applying IoT technology to the field of agriculture^2,3^. It connects and integrates agricultural fields, crops, and farming equipment through technologies such as sensors, wireless communication, cloud computing, and data analysis. Agriculture IoT typically involves diverse sensors and devices that enable real-time and precise data collection, as well as automated and intelligent agricultural production management. The importance of Agriculture IoT in the agricultural sector is evident as it can enhance agricultural productivity, conserve resources and reduce costs, improve the quality and safety of agricultural products, and provide decision support for farmers^4,5^. The integration of agricultural IoT with 5G/6G networks holds the promise of further amplifying these advantages, facilitating the implementation of more sophisticated services such as real-time pest and disease prediction, smart irrigation, and automated crop management.

The application scope of Agriculture IoT is wide-ranging, including but not limited to soil moisture monitoring, weather condition monitoring, water quality monitoring, precision fertilization, automated irrigation, smart farming, and agricultural product traceability^6–8^. In terms of soil moisture monitoring, farmers can precisely control irrigation water volume and improve water resource utilization efficiency by monitoring changes in soil moisture. At the same time, Agriculture IoT can be used for weather condition monitoring, enabling timely understanding of weather changes and rational adjustment of agricultural production plans by monitoring factors such as temperature, humidity, and light intensity. In addition, Agriculture IoT can be used for crop growth monitoring, pest and disease early warning, smart irrigation, and precision fertilization^9^.

In this paper, a wide application area of agricultural iot is the monitoring of pear cultivation. By deploying sensor nodes in pear cultivation areas, key parameters such as pear tree growth and development, soil moisture, temperature, and light intensity can be monitored and collected in real-time^10^. These data can be transmitted to cloud platforms for centralized storage and analysis, providing valuable information and decision support for farmers and agricultural experts. Leveraging Agriculture IoT technology, monitoring of pear cultivation can achieve precise agricultural management. By continuously monitoring soil moisture and temperature, farmers can adjust irrigation and fertilization strategies promptly, ensuring pear trees grow in optimal environmental conditions. Simultaneously, monitoring light intensity can help farmers optimize the direction and distribution of pear tree growth, enhancing yield and quality.

In the specific pear production data monitoring, Agricultural Wireless Sensor Networks (AWSNs) are wireless sensor networks specifically designed for the agricultural sector and are a critical component of Agriculture IoT^11–13^. By utilizing wireless communication technology and sensor nodes, AWSNs connect and transmit data from physical entities such as pear cultivation environment, pear crops, and agricultural equipment. The design goal of AWSNs is to achieve real-time monitoring and data collection of agricultural environments and crop growth, providing accurate agricultural management and decision support. AWSNs possess characteristics such as self-organization and adaptability, where sensor nodes can automatically form networks and self-configure, adapting to different cultivation environments and requirements. Additionally, AWSNs are distributed and collaborative, enabling communication and collaboration among sensor nodes to collectively accomplish data collection and processing tasks. AWSNs also exhibit low power consumption and long lifespan characteristics, as sensor nodes are designed with energy-saving features, prolonging the system’s usability^14,15^.

**Figure 1:**
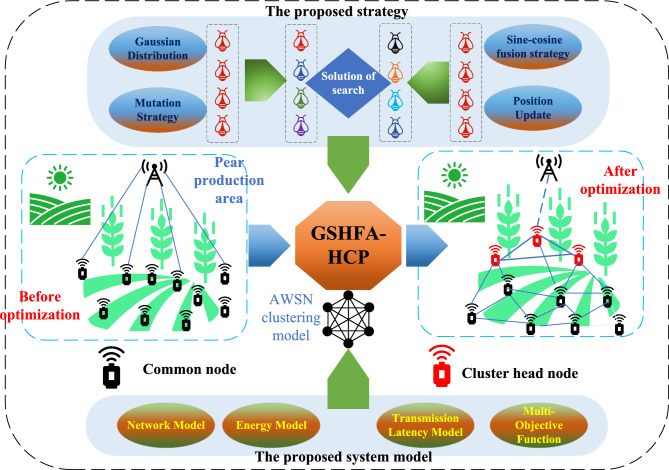
The core work framework of this paper.

In AWSN, especially in 5G/6G environments, the clustering operation is the process of organizing sensor nodes into clusters, where each cluster is led by a cluster head (CH) node^16–18^. The process can be represented by Fig. 1. The goal of the clustering operation is to optimize energy utilization and network performance. To achieve energy balance, the clustering operation typically adopts a round-robin selection strategy for choosing cluster heads, ensuring an even distribution of energy consumption. Additionally, through data aggregation techniques, similar data can be merged and compressed at the cluster head, reducing redundant data transmission and thereby decreasing energy consumption and network load^19^. The clustering operation plays a crucial role in AWSN by improving network performance and energy utilization efficiency, achieving goals such as energy-efficient utilization, optimized data transmission, and enhanced network performance. This provides reliable data support and decision-making basis for agricultural management, ultimately enhancing efficiency and quality in agricultural production.

In the context of AWSN, although hierarchical management can reduce energy consumption, clustering remains a challenging NP-hard problem with multiple optimization objectives, including latency, network lifespan, and energy balance^20,21^. Specifically, the election of CH nodes is a crucial step that involves considering factors such as remaining energy and distance^22,23^. The commonly used methods for solving clustering problems include traditional methods and heuristic methods.

Traditional methods have some limitations in the clustering operation of agricultural wireless sensor networks^24,25^. Traditional methods typically rely on fixed node selection strategies, which may lead to energy imbalance issues, with some nodes consuming excessive energy in a short period, resulting in shortened network lifetime. To overcome the limitations of traditional methods, current research tends to adopt heuristic algorithms for optimizing the clustering operation^26,27^. Heuristic algorithms are heuristic search methods based on experience and problem characteristics, searching for optimal solutions through iteration and optimization processes. In AWSN, heuristic algorithms can dynamically select cluster heads to achieve energy balance and prolong network lifetime. The widespread optimization of clustering operations using heuristic algorithms can improve the performance and efficiency of AWSN, providing more accurate and reliable data support for agricultural management.

To address this issue, this paper proposes a metaheuristic-based clustering protocol that combines Gaussian-mutated strategy and sine-cosine hybrid techniques. Through this approach, we aim to enhance the global search capability of clustering solutions, reduce the risk of falling into local optima, and achieve more efficient and balanced energy consumption in AWSN. The following section details the contributions of this paper.

1. A new AWSN multi-target clustering model is proposed, which calculates the latency according to the time of receiving and sending packets by sensor nodes. More importantly, factors such as node energy, node degree, average distance to neighbors, and delay were comprehensively considered as evaluation indicators, and a multi-objective weight matching method was introduced to meet the needs of the actual production scene of fragrant pear. It provides a more accurate and reasonable cluster-head selection scheme for wireless sensor networks. This improved scheme is not possible in most single factor clustering algorithms.
2. Under the premise of contribution (1), a high-performance clustering protocol (GSHFA-HCP) based on Gaussian mutation strategy and sine-cosine fusion strategy is proposed. Through the combination of the two strategies, the diversity of firefly population and the quality of approximate solutions are enhanced, and the convergence rate of GSHFA-HCP is significantly improved.
3. Finally, four popular schemes are selected as comparison protocols, and three experimental scenarios are designed to verify the performance of GSHFA-HCP. From the aspects of network life, energy consumption, throughput and transmission delay, the five clustering protocols are compared and analyzed in order to make the advantages of the proposed protocols convincing.

The overall structure of this paper is arranged as follows. “Introduction” section provides an introduction to Agriculture IoT and the research background of AWSN clustering problems. In “Related work” section, related research on clustering protocols is discussed. “Multi-objective clustering system model” section introduces the proposed novel multi-objective clustering model. The fourth section presents an AWSN clustering protocol based on the Gaussian mutation sine-cosine hybrid firefly algorithm. Second 5 describes a series of experiments that demonstrate the significant advantages of GSHFA-HCP in improving network lifetime, throughput, reducing energy consumption, and transmission delay. Finally, “Conclusion” section provides a summary and conclusion.

## Related work

On the basis of reviewing the existing literature, this paper summarizes the typical traditional clustering scheme, multi-factor clustering scheme and heuristic search mechanism in WSNs to better demonstrate the motivation of the protocol proposed in this paper. Then, summary of the surveyed work is given in Table 1.

**Table 1:** Summary of the surveyed work.

Literatures	Year	Methods	Characteristics
Abdurohman, Supriadi and Fahmi	2020	ME-LEACH	Focus on ensuring end-to-end secure and saving network energy
Hassan, Shah, Habeb, Othman and Al-Mhiqani	2020	IEECP	Improve the cluster structure and extend the network lifetime
Anitha et al.	2021	VC-SCCA	Adopt a newly developed balancing optimization algorithm and a multi-hop routing strategy
Heidari, Movaghar, Motameni and Barzegar	2022	GA-BOA	Use genetic algorithms for clustering and employed a balancing optimization algorithm to select the optimal path
Mehra, Doja and Alam	2019	E-CAFL	Improve clustering algorithm by leveraging fuzzy logic to enhance clustering efficiency
Wang, Gao, Wang, Sangaiah and Lim	2019	APSA	Studty an adaptive clustering technique based on affinity propagation
Dattatraya and Rao	2022	GSO-FOA	Select the optimal CH nodes by combining the glowworm swarm optimization and the fruitfly optimization algorithm
Mittal, Singh, Salgotra and Sohi	2019	FLGWO	Design a threshold-sensitive energy-efficient clustering protocol based on fuzzy logic extension of the grey wolf optimization algorithm
Cai, Geng, Wu, Wang and Wu	2020	BAT-LEACH	Improve the low energy adaptive clustering hierarchy protocol by a unified heuristic bat algorithm for optimizing
Mahajan and Badarla	2021	NICLCP	Design a natural-inspired cross-layer clustering protocol for dense monitoring application scenarios

### Literature review

In recent years, research in WSNs has shifted its focus towards energy optimization and scalability in cluster routing strategies. Reference^28^ proposed the end-to-end secure low energy adaptive clustering hierarchy (ME-LEACH) algorithm to enhance the lifespan of WSNs. Furthermore, an end-to-end secure low energy adaptive clustering hierarchy (E-LEACH) protocol has been developed to address the issue of high energy consumption. Existing clustering protocols have limitations in their cluster structures, which affect their efficiency. To tackle this problem, Ref.^29^ developed the improved energy-efficient clustering protocol (IEECP) with the aim of prolonging the lifespan of IoT devices based on WSNs.

However, the computational requirements of these clustering methods increase dramatically with the scale, making them impractical for application in AWSNs. AWSNs typically consist of a large number of sensor nodes that require frequent communication and data processing. These clustering methods encounter computational resource limitations when dealing with large-scale networks. This is attributed to the significant increase in time and space complexity of clustering methods as the number of sensor nodes grows, exceeding the available computational resources.

Reference^30^ introduced a new algorithm called voronoi-clustering secure context cryptographic algorithm (VC-SCCA), which combines voronoi techniques with cryptography and focuses on clustering applications. The algorithm was compared with two existing methods in terms of energy consumption, data transmission efficiency, network lifetime, and the performance of encryption and decryption processes. The study also emphasized how, after data collection, cluster heads efficiently transmit data to the base station by adopting a newly developed balancing optimization algorithm and a multi-hop routing strategy. Reference^31^ focused on utilizing genetic algorithms for clustering and employed a balancing optimization algorithm to select the optimal path between CH and the base station, aiming to reduce energy consumption in WSNs. They designed a robust balancing clustering method where selected nodes serve as cluster heads. Reference^32^ investigated an enhanced clustering algorithm that utilizes fuzzy logic in WSNs. They developed an improved clustering algorithm called E-CAFL, which optimizes the existing CAFL protocol by leveraging fuzzy logic to enhance clustering efficiency. Reference^33^ explored an adaptive clustering technique for Wireless Sensor Networks based on affinity propagation. They proposed the APSA clustering method, which utilizes the affinity propagation mechanism to enhance clustering functionality in WSNs.

These clustering methods have not adequately considered all the key factors that affect the actual performance of AWSNs, which may impose certain limitations and constraints in practical applications. In the actual usage of AWSNs, in addition to considering the energy levels and communication distances of sensor nodes, numerous other crucial factors need to be taken into account, such as node density and data transmission latency. These factors are of paramount importance for the performance and reliability of AWSNs. Due to the insufficient modeling and optimization of these key factors in these clustering methods, performance degradation or energy imbalance issues may arise in practical usage scenarios.

Reference^34^ innovatively developed a cluster head selection model to maximize the lifespan and energy efficiency of WSNs. The study introduced a novel algorithm, the fitness-based glowworm swarm and fruitfly algorithm, which combines the glowworm swarm optimization and the fruitfly optimization algorithm to select the optimal CH nodes in WSNs. Additionally, efficient CH selection algorithms and optimized routing strategies are crucial for designing effective solutions for large-scale networks, considering the limitations of cluster-based hierarchical network approaches. In this context, Ref.^35^ proposed a threshold-sensitive energy-efficient clustering protocol based on fuzzy logic extension of the grey wolf optimization algorithm to extend the stable operational period of the network. Furthermore, Ref.^36^ investigated a unified heuristic bat algorithm for optimizing the low energy adaptive clustering hierarchy protocol. They proposed a unified heuristic bat algorithm specifically for optimizing the election process of cluster heads. Lastly, Ref.^37^ addressed the dense deployment of WSNs for regular monitoring of environmental conditions in farms and proposed a natural-inspired cross-layer clustering protocol. This novel protocol aims to significantly improve the network lifespan and is suitable for dense monitoring application scenarios.

### Motivation

These heuristic based methods can partially solve the clustering problem in AWSNs. However, due to their tendency to converge prematurely and their difficulty in getting rid of local optimality, these methods make it difficult to find superior clustering solutions. Therefore, based on the above existing problems, the motivation of this paper is to design an effective heuristic clustering method to meet the actual needs of AWSNs. Firstly, a multi-objective optimization mechanism is formed by considering node energy, node degree, node average distance and data transmission delay comprehensively. Then, Gaussian mutation and sine cosine firefly algorithm are proposed to accelerate the convergence of clustering protocol and improve the overall optimization performance of GSHFA-HCP. The core working framework of this article is shown in Fig. 1.

## Multi-objective clustering system model

### Problem formulation

In an AWSN, the clustering problem can be defined as the partitioning of network nodes into multiple clusters, each cluster being led by a CH node. The CH is responsible for collecting data from member nodes within the cluster and aggregating the data before transmitting it to BS. The objective of this problem is to minimize energy consumption, prolong the network’s lifetime, and ensure effective data transmission.

Typically, an AWSN consists of *N* sensor nodes, represented as the set $S = {s_1, s_2,..., s_N}$. The network is divided into *K* clusters, with each cluster $C_k$ having a Cluster Head, denoted as $CH_k$. Each node $s_i$ belongs to exactly one cluster $C_k$, and the union of all clusters, denoted as $\bigcup _{k=1}^K C_k$, equals the set *S*. Additionally, the intersection between any two clusters $C_i$ and $C_j$ is empty, i.e., $C_i \cap C_j = \emptyset$, for all $i \ne j$.

### Network model

**Figure 2:**
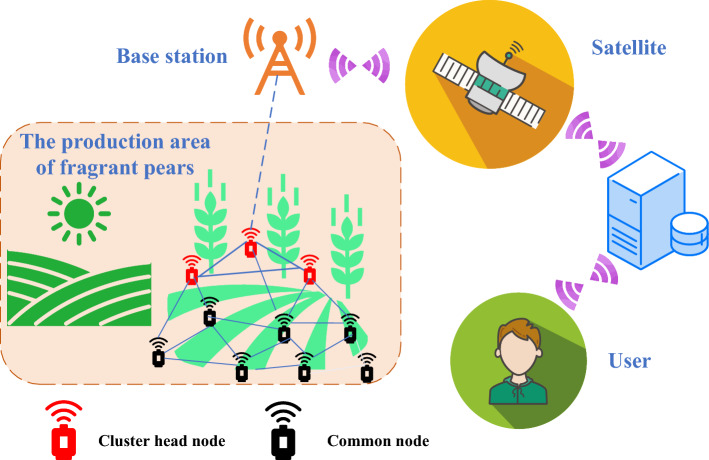
Network model architecture diagram.

Within the scope of the study based on AWSN monitored by fragrant pears, this study considers a network consisting of *N* nodes that are uniformly distributed within a rectangular monitoring area, with the base station also positioned within this area. In the context of AWSN, the core objective of clustering protocols is to enhance the overall performance of the network. Specifically, each node *i* in the network possesses a neighborhood set $N_i$, which represents the set of neighboring nodes, with $\vert N_i \vert$ quantifying the number of adjacent nodes to node *i*. Furthermore, to simplify the model and analysis, this research assumes a maximum communication distance of *distmax* for each node. The model and concepts described in this section are precisely defined in Table 2 to facilitate readers’ better understanding and tracking of the subsequent discussions and analyses in this paper. The network model is shown in Fig. 2.

**Table 2:** Symbol definition table.

Character	Description
*N*	Total number of nodes in the network
$N_i$	The neighborhood set of node *i*
$\vert N_i \vert$	The number of neighboring nodes of node *i*
*s*	Number of data packets sent by nodes
*dist*	The distance at which a node sends data packets
*Etrx*(*s*, *dist*)	Energy consumption of the node when transmitting *s* bits of data to a target node located at a distance of *dist*
*Erex*(*s*)	Energy consumption of the target node when receiving *s* bits of data
$E_{elec}$	Energy consumption of the circuit when the node receives or transmits 1 bit of data
$\xi _{fs}$	Communication energy coefficient for free space transmission
$\xi _{mp}$	Communication energy coefficient for multipath fading
*Etot*(*s*)	Total energy consumption of a node during the entire communication phase
*messageNum*	Total number of data packets
$\phi$	Network latency factor
*transDist* , *transRate*	The distance and speed of data packet transmission
*size*	Size of the data packets
*F*(*i*)	Fitness function for node *i*
*E*(*i*)	Remaining energy of a relay node
*Einit*	Initial energy of the node
*S*(*i*)	Total number of neighboring nodes within the communication radius of node *i*
*D*(*i*, *j*)	Distance from node *i* to its neighboring node *j*
*R*	Communication radius of the node
$\delta$	Delay impact parameter
$f_{eng} (i)$, $f_{deg} (i)$, $f_{dis} (i)$, $f_{del} (i)$	Objective function of network energy, node degree, distance, and latency, respectively
$\tau _1$, $\tau _2$, $\tau _3$, $\tau _4$	Weights for network energy, node degree, distance, and latency, respectively

Furthermore, we make the following assumptions regarding the network infrastructure model used by the proposed protocol:

1. Each node is uniquely identified by an ID.
2. All nodes have the same initial energy and can send sensed data to the base station or other nodes.
3. The base station is stationary and has infinite energy and other resources.
4. Nodes can communicate directly with each other, meaning that every node can directly communicate with all other nodes.
5. All nodes are homogeneous, and sensor nodes are similar in functionality and performance.

### Energy model

**Figure 3:**
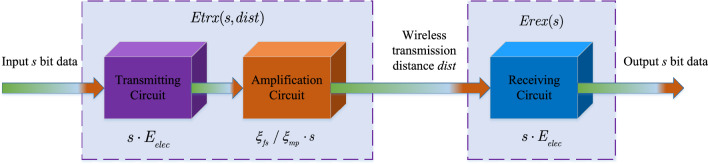
Energy model of AWSN.

The energy consumption of nodes in wireless sensor networks primarily occurs during sensing, processing, and communication, with the communication module accounting for over 90% of the total energy consumption. Therefore, this paper disregards the energy consumption during sensing and processing states and focuses solely on the communication energy consumption of nodes. To analyze this, we adopt a classical wireless communication energy model, considering both data reception and transmission. The network energy consumption model is structured as shown in Fig. 3.

Assuming that a sensor node transmits *s* bits of data to a target node located at a distance of *dist* meters. The energy consumed by the node can be represented by the following equation.

1 $$\begin{aligned} Etrx(s,dist) = \left\{ {\begin{array}{*{20}{c}} {s \cdot {E_{elec}} + s \cdot {\xi _{fs}} \cdot {dist^2}}&{}{dist \le {dist_0}}\\ {s \cdot {E_{elec}} + s \cdot {\xi _{mp}} \cdot {dist^4}}&{}{dist > {dist_0}} \end{array}}. \right. \end{aligned}$$

The energy consumption relationship for a sensor node receiving *s* bits of data is as follows:

2 $$\begin{aligned} Erex(s) = s \cdot {E_{elec}}, \end{aligned}$$

where *Etrx*(*s*, *dist*) represents the energy consumption of the node when transmitting *s* bits of data to a target node located at a distance of *dist*. *Erex*(*s*) represents the energy consumption of the target node when receiving *s* bits of data. $$E_{elec}$$ denotes the energy consumption of the circuit when the node receives or transmits 1 bit of data. $$dist_0 $$represents the communication threshold between nodes. When the distance is less than the threshold, the network energy consumption follows the free space channel model, $$\xi _{fs}$$ is the communication energy coefficient for free space transmission. When the distance is greater than or equal to the threshold, the network energy consumption follows the multipath fading channel model, $$\xi _{mp}$$ is the communication energy coefficient for multipath fading.

The total energy consumption of a node during the entire communication phase, denoted as *Etot*(*s*), is the sum of the energy consumption for data reception and transmission, and can be expressed as follows.

3 $$\begin{aligned} Etot(s) = Etrx(s,dist) + Erex(s). \end{aligned}$$

### Transmission latency model

To ensure the effectiveness of the AWSN clustering protocol in facilitating efficient data transmission and processing, it is crucial to focus on the performance of transmission latency. Excellent transmission latency is vital for timely data acquisition in agricultural monitoring endpoints, as it aids in enabling more effective selection of technological means. Furthermore, considering the broad applicability and universality of the protocol, and to ensure a more accurate assessment of latency in practical agricultural applications, the average data transmission latency during the AWSN clustering process is calculated as the ultimate AWSN latency metric.

4 $$\begin{aligned} Qdel = \frac{\sum _{i=1}^{messageNum}(1/transRate) \cdot (\phi \cdot transDist +size)}{messageNum}, \end{aligned}$$

where *messageNum* represents the total number of data packets, $\phi$ is the network latency factor, *transDist* and *transRate* denote the distance and speed of data packet transmission, and *size* represents the size of the data packets.

### Multi-objective evaluation function

Upon completion of node deployment, during the network initialization phase, nodes exchange information with their neighboring nodes until all nodes receive the ID, position, and energy information sent by their neighbors. This marks the beginning of the cluster setup phase in the GSHFA-HCP protocol. The selection of cluster heads is based on four factors: energy, distance, node density, and transmission quality. Let F(i) denote the fitness function for node i to compete for the cluster head position. To define the fitness function, we primarily consider four metrics: remaining energy of the node, node degree, average distance from the node to its neighboring nodes, and transmission delay.

During the operation of the network, in each round, cluster heads (CHs) need to receive data transmitted by their cluster members and forward it to the base station (BS) after fusion. This process consumes a significant amount of energy for the cluster heads. To prevent cluster heads from being unable to perform their tasks due to energy depletion, they need to be selected as nodes with higher energy. The computation of the energy objective function is shown in Eq. (5).

5 $$\begin{aligned} f_{eng} (i) = \frac{E(i)}{E_{init}}, \end{aligned}$$

where *E*(*i*) represents the remaining energy of a relay node, and $E_{init}$ denotes the initial energy of the node.

Node degree refers to the number of neighboring nodes within the communication range. When a regular node joins a specific cluster, it should choose to join the cluster headed by a neighboring node. Therefore, the cluster head needs to be selected as a node with higher node density and more neighboring nodes. This ensures that the selected cluster head has better centrality within the cluster. The expression for this is given by Eq. (6).

6 $$\begin{aligned} f_{deg} (i) = \frac{S(i)}{N}, \end{aligned}$$

where *S*(*i*) represents the total number of neighboring nodes within the communication radius of node *i*, and *N* denotes the total number of nodes in the network.

As energy consumption is primarily concentrated during the transmission process, reducing the transmission distance is a key factor in reducing energy consumption. Therefore, in addition to selecting nodes with a higher number of neighboring nodes, cluster heads (CHs) should strive to minimize the average distance between neighboring nodes and the cluster head. This can be expressed by Eq. (7).

7 $$\begin{aligned} f_{dis} (i) =1- \frac{\sum _{j=1}^{neigh} D(i,j)}{S(i) \cdot R}, \end{aligned}$$

where *D*(*i*, *j*) represents the distance from node *i* to its neighboring node *j*, and *R* denotes the communication radius of the node.

In AWSN, latency refers to the time it takes for data generated at a sensor node to reach the destination node. Latency is a crucial performance metric that directly affects the degree to which AWSN meets real-time requirements. Therefore, the selection of CH nodes must consider the performance of latency, which can be computed using Eq. (8).

8 $$\begin{aligned} f_{del} (i) =\delta \cdot Qdel. \end{aligned}$$

The parameter $\delta$ represents the delay impact parameter.

Lastly, considering all the aforementioned parameters, the objective function F for all surviving nodes is defined as shown in Eq. (9).

9 $$\begin{aligned} min F_{eval} =\tau _1 \cdot f^*_{eng} + \tau _2 \cdot f^*_{deg} + \tau _3 \cdot f^*_{dis} + \tau _4 \cdot f^*_{del} , \end{aligned}$$

10 $$\begin{aligned} s.t.\left\{ \begin{array}{l} 0 \le f_{eng}^* \le 1\\ 0 \le f_{deg}^* \le 1\\ 0 \le f_{dis}^* \le 1\\ 0 \le f_{del}^* \le 1\\ \tau _1 + \tau _2 + \tau _3 +\tau _4=1\\ \end{array} \right. , \end{aligned}$$

where $f_{eng}^*$, $f_{deg}^*$, $f_{dis}^*$ and $f_{del}^*$ respectively represent the normalized values of the four indicators. $\tau _1$, $\tau _2$, $\tau _3$, and $\tau _4$ represent the weights for network energy, node degree, distance, and latency, respectively.

In this study, the clustering process involves the utilization of a fitness function that comprehensively considers multiple key attributes of the nodes, including energy level, spatial distance, node density, and transmission quality. This comprehensive evaluation approach enables a more holistic assessment of the performance and adaptability of the nodes. Among these considerations, node density and spatial distance are regarded as constant factors during the clustering process. In contrast, the energy level of the nodes plays a crucial role in the selection of CH nodes. This is because assigning higher importance to the energy parameter in the fitness function helps prevent specific nodes from being repeatedly chosen as cluster heads, thus avoiding premature depletion of these nodes. Therefore, in the fitness function F, the coefficient a for the energy parameter should generally be set not lower than the coefficients for other parameters to ensure that the energy factor receives appropriate priority consideration in the selection of cluster heads.

## GSHFA-HCP process for solving clustering problems

In addressing the clustering problem in the data transmission process of AWSN for monitoring pear crops, GSHFA-HCP utilizes the firefly’s bioluminescence and mating behavior for solution search. Essentially, GSHFA-HCP is based on a random search algorithm that explores different possibilities through trial and error. During the search process, other behavioral characteristics of fireflies are ignored, and only the features that are beneficial for problem-solving are extracted. This is manifested in three assumptions of the algorithm,In addressing the clustering problem in the data transmission process of AWSN for monitoring pear crops, the GSHFA-HCP algorithm utilizes the firefly’s bioluminescence and mating behavior for solution search. Essentially, GSHFA-HCP is based on a randomized search algorithm that explores different possibilities through trial and error. During the search process, other behavioral characteristics of fireflies are disregarded, and only the characteristics that are relevant for problem-solving are extracted. Specifically, this algorithm is based on three assumptions:

1. Fireflies are neutral, and their gender is no longer considered. The attractiveness between fireflies is solely determined by the brightness of their bioluminescence and is independent of other factors.
2. The attractiveness of a firefly is closely related to the brightness of its bioluminescence. A higher brightness corresponds to a stronger attractiveness, while a lower brightness corresponds to a weaker attractiveness.
3. The brightness of a firefly’s bioluminescence is uniquely determined by the objective function value of the algorithm and is independent of other factors. The core idea of the algorithm is that due to the different positions of each firefly, they have different objective function values, which in turn determine their brightness and attractiveness. Fireflies with higher brightness attract those with lower brightness to move towards them, updating their positions. In each iteration, every individual firefly moves accordingly, eventually converging to the best position within the group and obtaining the optimal solution.

### Encoding and population initialization

In GSHFA-HCP, as the primary step of the firefly algorithm, appropriate encoding and random generation of initial positions provide diversity and global search capabilities to the algorithm, laying a solid foundation for the subsequent search process. Individuals are typically represented as real-valued vectors, with each dimension corresponding to a feature or variable of the problem. By randomly generating initial positions, each firefly individual has the opportunity to represent a different potential solution. The importance of this step lies in the fact that a well-initialized firefly population can effectively enhance the efficiency and effectiveness of the search process. Each firefly represents an individual encoded as a real number. However, during the search for clustering solutions, these individuals need to be converted into binary encoding. The conversion method is as follows: the nodes corresponding to the *cnum* highest numerical values are designated as CH while the nodes corresponding to other positions are designated as CM. The population is represented as follows:

11 $$\begin{aligned} \begin{array}{l} {Z_{(M,N)}} = \left[ {\begin{array}{*{20}{c}} {{z_{1,1}}}&{}{{z_{1,2}}}&{} \cdots &{}{{z_{1,N - 1}}}&{}{{z_{1,N}}}\\ {{z_{2,1}}}&{}{{z_{2,2}}}&{} \cdots &{}{{z_{2,N - 1}}}&{}{{z_{2,N}}}\\ \cdots &{} \cdots &{}{{z_{m,n}}}&{} \cdots &{} \cdots \\ {{z_{M - 1,1}}}&{}{{z_{M - 1,2}}}&{} \cdots &{}{{z_{M - 1,N - 1}}}&{}{{z_{M - 1,N}}}\\ {{z_{M,1}}}&{}{{z_{M,2}}}&{} \cdots &{}{{z_{M,N - 1}}}&{}{{z_{M,N}}} \end{array}} \right] \\ ({z_{m,n}} \in [1,N],n \in [1,N],m \in [1,M]) \end{array}, \end{aligned}$$

where ${Z_{(M,N)}}$ represents the firefly population, *M* represents the population size, and *N* represents the number of sensors.

### Brightness evaluation

In each solution, this study adopts a strategy of selecting the *cnum* nodes with the highest values from all the nodes as CH nodes, while considering the remaining nodes as regular nodes. Subsequently, by applying formula (9), this research calculates the fitness value of the population, which serves as the brightness of the firefly population. The algorithm updates the population brightness and the best solution at the beginning of each iteration. Specifically, the algorithm identifies the firefly with the highest brightness in the current population, and its corresponding position is recognized as the optimal solution. This process reflects the optimization of the fitness evaluation method for the population and an effective strategy for searching for the optimal solution in a dynamic environment.

### Attraction update

Assuming a firefly population with a total number of *M* individuals, where each firefly represents a candidate solution, the $i_{th}$ firefly can be represented as $Z_i = (z_{i1}, z_{i2},..., z_{in})$, where *n* denotes the dimensionality of the problem to be solved. The brightness of each firefly is defined by the following formula.

12 $$\begin{aligned} L=L_0 \cdot e^{- \xi \cdot d_{ij}^2}. \end{aligned}$$

In this equation, $L_0$ represents the initial light intensity of a firefly, $\xi$ denotes the light absorption factor, and $d_{ij}$ represents the distance between individuals *i* and *j*. The specific calculation method can be expressed by the following formula.

13 $$\begin{aligned} d_{ij}=\vert \vert Z_i-Z_j \vert \vert =\sqrt{\sum _{k=1}^d (z_{ik}-z_{jk})^2}. \end{aligned}$$

Due to the close relationship between brightness and attraction, fireflies located at different positions exhibit different levels of attraction towards each other. The definition of the attraction function for these fireflies is as follows:

14 $$\begin{aligned} H(d_{ij})=H_0 \cdot e^{- \xi \cdot d_{ij}^2}. \end{aligned}$$

Here, $H_0$ refers to the attraction when the distance between individuals is zero.

### Integration of sine–cosine position update

In the process of updating the positions of fireflies, the principles of the sine–cosine algorithm are incorporated, along with the introduction of a non-linear sine learning factor, which greatly enhances the search speed for solutions. During the initial stage of the search, the learning factor typically has a larger value, which facilitates the algorithm in conducting a global search and extensively exploring the search space for potential optimal solutions. Conversely, during the later stage of the search, the learning factor typically has a smaller value, enabling the algorithm to focus more on local search to exploit and uncover potential local optimal solutions within the search space, thereby improving the optimization accuracy of the algorithm. This dynamic adjustment strategy of the learning factor allows the algorithm to exhibit better search performance and higher search efficiency in different search stages. Consequently, the updating approach of the firefly algorithm becomes more flexible, enabling it to better adapt to different search spaces and enhance the search efficiency and accuracy of the algorithm. The formula for the learning factor eta and the firefly formula incorporating the sine–cosine algorithm are presented as follows.

15 $$\begin{aligned} \eta =\eta _{\min} + (\eta _{\max} -\eta _{min}) \cdot \sin(\frac{t \cdot \pi }{maxgen}), \end{aligned}$$

where $\eta _{\min}$ and $\eta _{\max}$ represent the upper and lower bounds of the learning factor, respectively, and *t* and *maxgen* denote the current iteration and the maximum number of iterations. The position update formula for the movement between firefly $Z_i$ and firefly $Z_j$ is given as follows.

If the brightness of firefly $Z_i$ is brighter than that of firefly $Z_j$, firefly $Z_i$ will be attracted by firefly $Z_j$. Otherwise, firefly $Z_j$ will attract firefly $Z_i$. Specifically, the following search strategy is employed.

16 $$\begin{aligned} Z_i(t+1){} & {} = (1-\eta ) \cdot Z_i(t)+ \theta \cdot (Z_j(t)-Z_i(t))\nonumber \\{} & {} \quad + \eta \cdot \sin(r_1) \cdot \vert r_2 Z_b(t) -Z_j(t) \vert , \end{aligned}$$

17 $$\begin{aligned} Z_j(t+1){} & {} =(1-\eta ) \cdot Z_j(t)+ \theta \cdot (Z_i(t)-Z_j(t))\nonumber \\{} & {} \quad + \eta \cdot \cos(r_1) \cdot \vert r_2 Z_b(t) -Z_i(t) \vert , \end{aligned}$$

where $Z_i(t+1)$ represents the updated position of firefly *i*, and $Z_i(t)$ and $Z_j(t)$ refer to the positions of firefly *i* and *j* in the $t-th$ generation, respectively. $r_1$ and $r_2$ are two distinct random numbers.

### Gaussian distribution-based mutation strategy

During the search process, the movement of fireflies can be limited by local optima due to their reliance on brightness and attractiveness. To avoid getting trapped in local optima, this paper proposes a mutation operation for the firefly population based on Gaussian distribution. Gaussian distribution is a commonly used probability distribution function that has been widely applied in optimization and design in engineering applications, as it exhibits favorable promoting effects. By introducing random perturbations following Gaussian distribution, the diversity of the population can be increased, thereby preventing fireflies from prematurely converging to local optima and improving the global search capability and optimization accuracy of the algorithm.

Specifically, to avoid being trapped in local optima, a stagnation detection strategy is employed. When the global best value of the population remains unchanged for a consecutive $g_0$ iterations, it indicates that the population has reached a local optimum region and the evolution process has stagnated. To enhance the diversity of the population and help the algorithm escape from local optima, Gaussian distribution is introduced to perform mutation operations on the population. The random variables generated by Gaussian distribution possess good randomness and continuity, enabling effective perturbations in the search space and facilitating the escape from local optima.

Through this approach, the search capability of GSHFA-HCP is further enhanced, enabling the algorithm to exhibit better global search and local exploration abilities, and better adapt to complex optimization problems. The probability density function of the Gaussian distribution is given by the following formula.

18 $$\begin{aligned} f(x)=\frac{1}{\sqrt{2 \pi } \sigma } e^{- \frac{(x-\mu )^2}{2 \sigma ^2}}, \end{aligned}$$

where $\sigma$ represents the variance of the Gaussian distribution, and $\mu$ represents the expectation.

19 $$\begin{aligned} Z_i=Z_i+N(0,1). \end{aligned}$$

Specifically, all firefly individuals in the population are sorted according to the magnitude of their objective function values. The top $10\% \times n$ fireflies, which are the best individuals, are selected as the elite group of the population. These elite fireflies are then used to update the states of the bottom $10\% \times n$ firefly individuals in terms of their ranking. Additionally, Gaussian mutation is applied to the updated firefly individuals to increase the diversity of the population and prevent premature convergence to local optima. Through this strategy, we can effectively utilize the excellent individuals in the population, thereby improving the search efficiency and optimization accuracy of the algorithm, while avoiding premature convergence to local optima. The mutation formula can be expressed as formula (18).

Here, *N*(0, 1) represents a random vector that follows a Gaussian distribution with an expectation of 0 and a variance of 1.

### Termination criteria

The convergence of the algorithm is determined by monitoring the changes in the objective function values. If the changes remain small within a certain number of iterations, specifically not exceeding the threshold $l_0$, it can be considered that the algorithm has converged, and the iteration process is then terminated.

**Figure 4:**
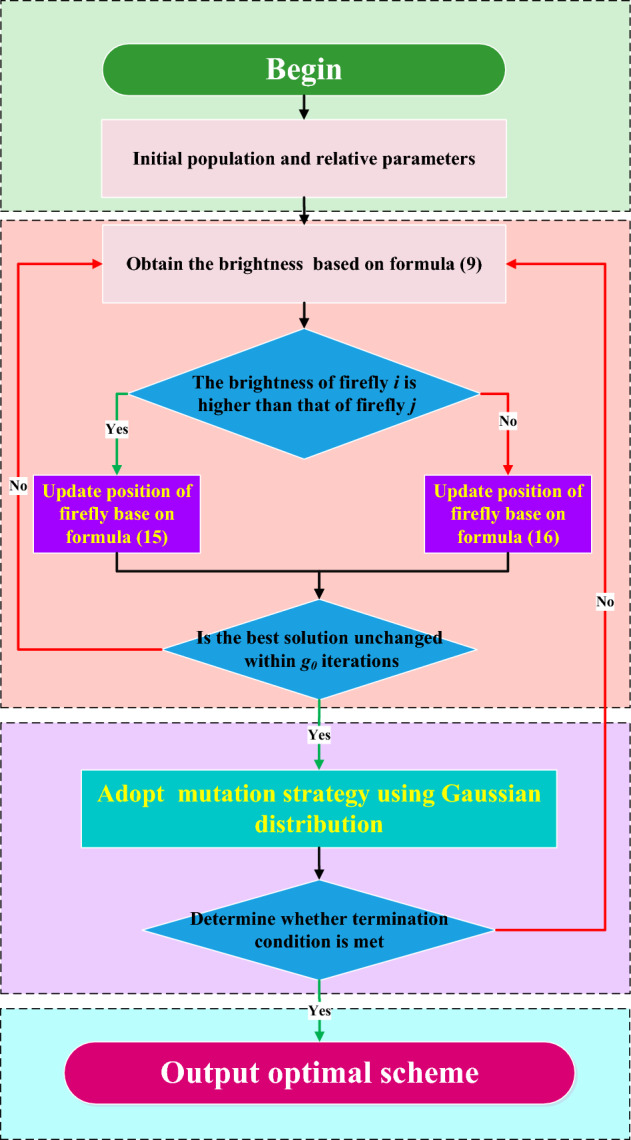
Overall flow of GSHFA-HCP.

### The overall process of the proposed GSHFA-HCP protocol

After introducing the core ideas and basic operations of the algorithm, the described operations can be divided into the following main steps, outlined as follows:

1. *Parameter setting* Set the relevant parameters of the algorithm, including the light absorption coefficient, initial light intensity, and self-attraction.
2. *Population initialization* Generate random numbers for each firefly in the population, setting the upper and lower limits of the random numbers to control them within an appropriate range.
3. *Brightness evaluation* Evaluate the brightness of each firefly by calculating the objective function value for individuals at different positions. To improve efficiency, typically the firefly individuals are sorted after comparing their brightness, facilitating brightness comparisons between fireflies.
4. *Attraction calculation* Assess the level of attraction between different individuals and utilize attraction to guide individual movement and interactions during the iterative process of the firefly algorithm.
5. *Updating firefly positions using the fusion of sine and cosine methods* Utilize the attraction between fireflies to update their positions using a position update formula. Then calculate the brightness of the updated individuals.
6. *Mutation based on Gaussian distribution* Apply Gaussian distribution sampling to the positions of each dimension of the firefly individuals, introducing small random perturbations to their positions within the search space.
7. If the change in positions still exceeds a threshold within a certain number of iterations, return to step 4 to update the positions and brightness of the fireflies, allowing the population to move by iterating again in a loop until convergence to the optimal position.

The general process of the GSHFA-HCP algorithm can be represented using a flowchart, as shown in Fig. 4.

## Experiment result and discussion

This study conducted simulation experiments using MATLAB to evaluate the performance of LUET^38^, DMaOWOA^39^, HFLFO^40^, and ARSH-FATI-CHS^41^ methods, as well as the proposed GSHFA-HCP algorithm, in addressing key issues such as network energy consumption, network lifetime, and network data transmission quality in AWSNs. To measure the performance of the proposed method, the study considered metrics such as the number of rounds with different proportions of node deaths, average remaining energy of nodes, throughput, and transmission delay.

In the GSHFA-HCP, the total number of fireflies was set to 50, with a maximum iteration count of 100. The attraction decay coefficient was set to 0.75, and the light intensity enhancement coefficient was set to 1. These parameters collectively determined the search capability and convergence speed of the firefly algorithm, ultimately influencing the efficiency and accuracy in finding the optimal solution.

For the experimental evaluation of clustering protocols in AWSNs, a total of 100 nodes were randomly and uniformly distributed within a monitoring area of 500 m by 500 m. Each node had a communication range of 30 m. The energy consumption model considered a transmission energy of 50 nJ/bit and a reception energy of 10 nJ/bit. The data packet size was set to 4000 bits the control packet size is set to 400 bits. The base station was positioned at the center of the monitoring area. These parameter settings provided the necessary foundation for simulations and are utilized to assess the performance of clustering protocols in real-world agricultural monitoring scenarios. Specifically, the parameters are given in Table 3 in detailed.

**Table 3:** Expertiment setting table.

Character	Description
AWSN monitoring area	500 m $\times$ 500 m
Number of nodes	$\left\{ 160,200,260 \right\}$
Proportion of CH	$\left\{ 0.05,0.1,0.2 \right\}$
Node communication radius	70 m
Location of base station	(250, 250)
Data packet size	4000 bits
Control packet length	400 bits
$E_{elec}$	50 nJ/bit
$\xi _{fs}$	10 pJ/b/m$^{2}$
$\xi _{mp}$	0.0013 pJ/b/m$^{2}$
*Einit*	0.8 J
$\delta$	0.37
($\tau _1$, $\tau _2$, $\tau _3$, $\tau _4$)	(0.3, 0.3, 0.3, 0.1)
Maximum iteration count	100
Number of firefly	50
Step	1.0
Light absorption factor $\xi$	1
Attraction decay coefficient	0.75

### Node death rounds

**Figure 5:**
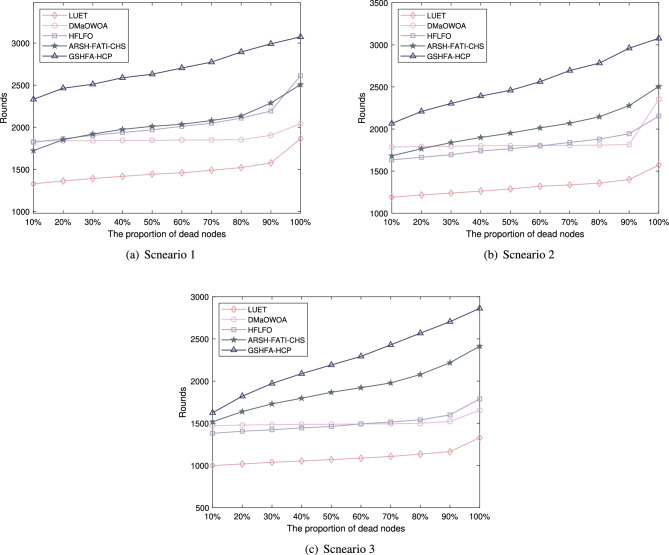
Death time of nodes with different proportions.

The study investigates the number of rounds in which a certain proportion of nodes die in a network, and the simulation results are presented in Fig. 5. From the simulation results, it is evident that LUET exhibits the earliest occurrence of node deaths at different proportions compared to the other methods. However, as the network scale increases, its performance deteriorates. This can be attributed to the inadequate consideration of key metrics such as node energy, node density, distance in the network, and data transmission quality in LUET.In small-scale scenarios, the performance of ARSH-FATI-CHS is comparable to DMaOWOA and HFLFO. However, in larger-scale AWSNs, the network lifetime of ARSH-FATI-CHS exceeds that of these two methods. Notably, in all considered scenarios, including small-scale, medium-scale, and large-scale scenarios, GSHFA-HCP demonstrates the longest node survival time, significantly outperforming the other comparative methods. For instance, in the small-scale scenario, the number of rounds with 10%, 50%, and 100% node deaths for GSHFA-HCP are 2332, 2630, and 3074, respectively, while the corresponding values for ARSH-FATI-CHS are 1723, 2011, and 2507. This superiority of GSHFA-HCP can be attributed to its excellent comprehensive evaluation and assessment of network energy, node density, distance in the network, and data transmission quality. These findings provide substantial evidence for the effectiveness of the proposed GSHFA-HCP algorithm in terms of energy consumption.

### Network lifetime

The lifespan of a network is defined as the time from the beginning of its operation to the death of the last node in the network. The calculation formula can be expressed as follows.

20 $$\begin{aligned} \text {Network lifetime}=T_{\text {last node death}} - T_{\text {network begin}}, \end{aligned}$$

where $T_{\text {last node death}}$ is the time when the last node died, and $T_{\text {network begin}}$ is the time when the network started running.

**Figure 6:**
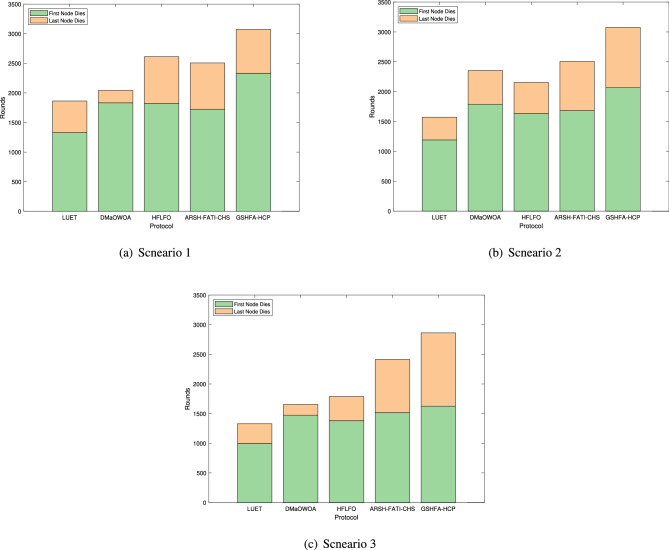
Overall liftetime of AWSN.

The network lifetime of LUET, DMaOWOA, HFLFO, ARSH-FATI-CHS, and GSHFA-HCP methods is depicted in Fig. 6. In the graph, each stacked column represents the number of rounds in which the last node dies, while the middle line indicates the number of rounds in which the first node dies. From scenario 1 in Fig. 6, it is evident that GSHFA-HCP significantly extends the network lifetime compared to LUET, DMaOWOA, HFLFO, and ARSH-FATI-CHS. Specifically, the last node in GSHFA-HCP dies after 991, 392, 402, and 536 rounds later compared to LUET, DMaOWOA, HFLFO, and ARSH-FATI-CHS, respectively. Similarly, it can be observed in the graph that GSHFA-HCP prolongs the network lifetime by 1176, 1027, 451, and 503 rounds relative to LUET, DMaOWOA, HFLFO, and ARSH-FATI-CHS, respectively. In comparison to other protocols, GSHFA-HCP exhibits an average improvement in network lifespan of 63.69%. This improvement can be attributed to the Gaussian mutation strategy employed in GSHFA-HCP, which enhances the diversity of the population and, consequently, improves the quality of the AWSN solution. As a result, GSHFA-HCP effectively reduces the energy consumption of individual nodes, leading to a significant extension of the AWSN network lifetime.

### Network energy

**Figure 7:**
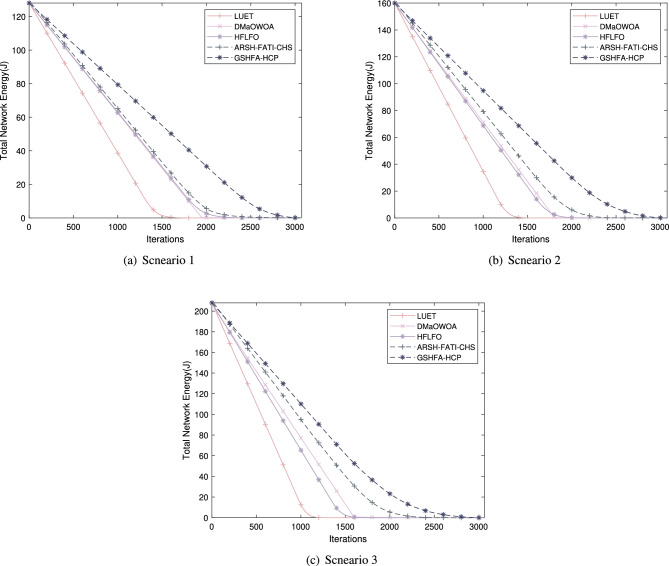
Network energy of AWSN.

Figure 7 presents a comparison of the average remaining energy of nodes among the five clustering methods. It can be observed from the graph that the proposed GSHFA-HCP exhibits significantly higher average remaining energy compared to the other four methods. Specifically, in comparison to the second-best performing ARSH-FATI-CHS method, GSHFA-HCP demonstrates a much slower rate of energy depletion in the network. For instance, in a large-scale AWSN scenario, after 1500 rounds, the overall network energy in GSHFA-HCP is 60.9956 J, while in ARSH-FATI-CHS, it is only 40.5286 J. This validates that GSHFA-HCP effectively reduces energy consumption and prolongs the network lifetime. The reason behind this improvement lies in the fact that the other methods suffer from suboptimal CH selection, leading to rapid energy depletion in the network, thereby affecting the network’s lifetime. In contrast, the GSHFA-HCP protocol optimizes the CH selection process effectively, which results in reduced energy consumption among the nodes in the network.

### Throughput

Throughput refers to the total number of data packets successfully transmitted to the base station within a specific time frame. The calculation formula can be expressed as follows.

21 $$\begin{aligned} \text {Throughput} = Pn_{succ}, \end{aligned}$$

where $Pn_{succ}$ is the number of successfully received packets within the simulation time.

**Figure 8:**
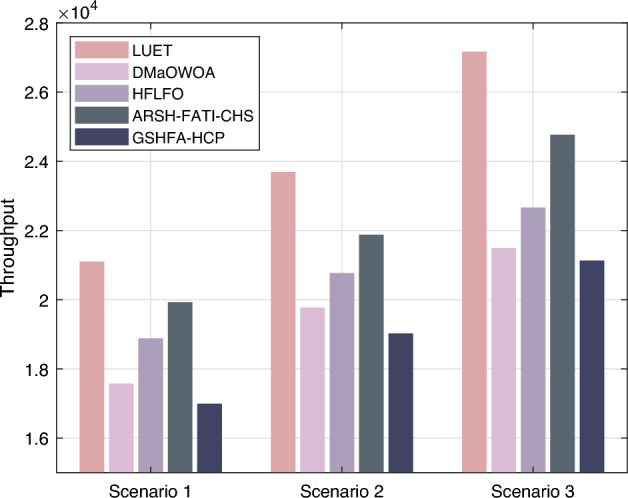
Throughput of AWSN.

Figure 8 illustrates the total number of data packets received by the base station for each clustering method. It can be observed that GSHFA-HCP receives a higher number of data packets compared to LUET, DMaOWOA, HFLFO, and ARSH-FATI-CHS. This difference is particularly evident in large-scale scenarios, where GSHFA-HCP receives a total of 2401 data packets, surpassing the performance of the second-best method, ARSH-FATI-CHS, by approximately 9.70%. Furthermore, GSHFA-HCP demonstrates an average throughput enhancement of 17.2% relative to alternative protocols. The reason behind this improvement can be attributed to the introduction of a novel fitness function in the GSHFA-HCP protocol. This enables nodes with superior overall performance indicators to have a higher probability of being selected as cluster heads. Moreover, GSHFA-HCP ensures that the obtained AWSN clustering solution is superior and better suited for the AWSN application scenario. Consequently, the protocol transmits the highest number of data packets among all the compared methods, indicating its higher energy efficiency compared to the other methods, which struggle to achieve similar performance.

### Transmission delay

Transmission delay is defined as the average time it takes for a packet to be sent from the source node to the base station. The calculation formula can be expressed as follows.

22 $$\begin{aligned} \text {Transmission Delay}=\frac{1}{Pn_{send}}\sum _{i=1} ^{Pn_{send}}(De_i+Pc_i), \end{aligned}$$

where $Pn_{send}$ is the total number of packets sent, $De_i$ is the transmission delay of the *i*-th packet, and $Pc_i$ is the processing delay of the *i*-th packet.

**Figure 9:**
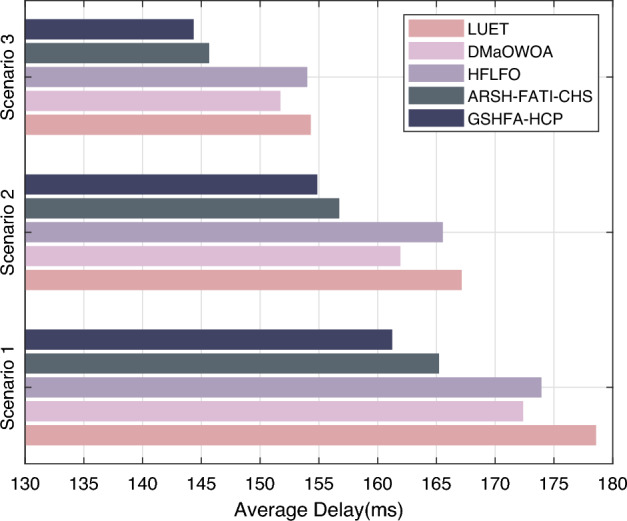
Transmission delay of AWSN.

Figure 9 shows the transmission delay of different protocols. The experimental comparison of clustering and routing protocols for AWSNs reveals significant performance differences in terms of network latency. LUET exhibits the poorest performance in terms of network latency due to its clustering strategy that is not well-suited for AWSN clustering problems, resulting in higher network delays. Although the ARSH-FATI-CHS protocol improves upon LEACH, it still suffers from higher network latency, especially in smaller-scale networks. In contrast, GSHFA-HCP shows significant progress in reducing network latency compared to the previous two protocols. In scenario 1, for instance, the network data transmission latencies of GSHFA-HCP, ARSH-FATI-CHS, and LUET are 178.54 ms, 165.18 ms, and 161.20 ms, respectively. GSHFA-HCP further reduces network latency, demonstrating better performance compared to ARSH-FATI-CHS, achieving the lowest network latency among all tested protocols. Moreover, the transmission latency of GSHFA-HCP is observed to decrease by an average of 19.56% compared to other protocols. This highlights the significant advantage of GSHFA-HCP in optimizing communication paths and reducing transmission delays. Overall, the experimental results indicate that as the number of network nodes increases, network latency gradually decreases, with GSHFA-HCP exhibiting outstanding performance in optimizing AWSN network latency. By optimizing key elements, an efficient and reliable solution for wireless sensor networks has been provided to meet the practical requirements of different scenarios. This paper proposes the GSHFA-HCP protocol, which combines Gaussian mutation strategy and sine–cosine hybrid strategy, achieving significant improvements in population diversity and solution quality. This greatly enhances the convergence speed of the algorithm, enabling it to search for high-quality clustering solutions for AWSNs. Consequently, it significantly reduces energy consumption in the network, effectively prolongs network lifetime, and ensures communication quality.

### Packet loss rate

The packet loss rate is defined as the complement of the ratio between the number of successfully transmitted data packets and the total number of attempted transmissions throughout the entire lifecycle. It represents the proportion of lost packets during the transmission process. The calculation formula is typically as follows.

23 $$\begin{aligned} \text {Packet Loss Rate} = \left( \frac{Pn_{loss}}{Pn_{send}}\right) \times 100. \end{aligned}$$

Here, $Pn_{loss}$ refers to the number of packets that did not successfully reach the destination during transmission, while $Pn_{send}$ represents the total number of packets attempted to be sent from the source.

**Figure 10:**
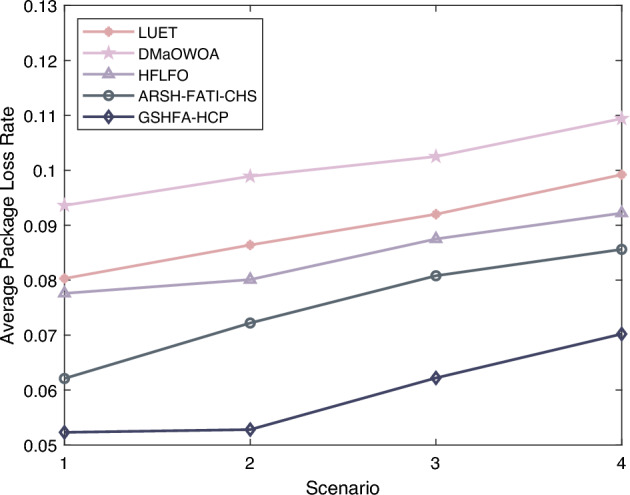
Packet loss rate of AWSN.

In the experimental study of clustering protocols in AWSNs, the packet loss rate of LUET, DMaOWOA, HFLFO, ARSH-FATI-CHS, and GSHFA-HCP was evaluated and the results are shown in Fig. 10. In scenario 1, LUET had a packet loss rate of 5%, DMaOWOA had 9.36%, HFLFO recorded 7.76%, ARSH-FATI-CHS had 6.21%, while GSHFA-HCP exhibited the lowest packet loss rate at 5.23%. In scenario 2, LUET’s packet loss rate increased to 8.64%, DMaOWOA increased to 9.89%, HFLFO was 8.01%, ARSH-FATI-CHS was 7.22%, and GSHFA-HCP maintained a lower rate of 5.28%. GSHFA-HCP consistently displayed a lower packet loss rate, and similar conclusions can be drawn in other experimental scenarios. GSHFA-HCP manifests an average reduction in packet loss rate of 35.78% in comparison with its counterparts, indicating its superior network stability. This is mainly due to the Gaussian mutation strategy, which enables the protocol to efficiently find the optimal clustering scheme, effectively organizing the network nodes into clusters, reducing management overhead and communication errors, and consequently lowering the packet loss rate.

## Conclusion

In this paper, a novel clustering model for AWSNs is proposed, taking into account factors such as node energy, node degree, average distance to neighbors, and latency. Additionally, a high-performance clustering protocol, GSHFA-HCP, is designed to meet the requirements of diverse operational environments. Furthermore, innovative enhancements to GSHFA-HCP are introduced by integrating Gaussian mutation and sine-cosine hybrid strategies. Finally, an efficient inter-cluster data transmission mechanism is devised based on the distances between nodes, remaining energy, and load. Experimental results demonstrate that the proposed GSHFA-HCP protocol effectively reduces network energy consumption, significantly prolongs network lifespan, and diminishes transmission latency.

In the future, we will transplant the experimental scene to the 3D environment for experiments, and consider the mobility of nodes, so as to study a new clustering scheme suitable for mobile scenes.

## Data Availability

The data that support the findings of this study are available from the corresponding author.

## References

[1] Quy, V. K., Nguyen, D. C., Van Anh, D. & Quy, N. M. Federated learning for green and sustainable 6g iiot applications. *Internet Things* **25**, 101061 (2024). 10.1016/j.iot.2024.101061

[2] Misra, N. N. *et al.* Big data, and artificial intelligence in agriculture and food industry. *IEEE Internet Things J.* **9**(9), 6305–6324 (2020). 10.1109/JIOT.2020.2998584

[3] Luo, T. *et al.* An innovative cluster routing method for performance enhancement in underwater acoustic sensor networks. *IEEE Internet Things J.* **1**, 1 (2024).

[4] Bashir, R. N., Bajwa, I. S. & Shahid, M. M. A. Agriculture iot: Emerging trends, cooperation networks, and outlook. *IEEE Internet Things J.* **7**(5), 4464–4472 (2020). 10.1109/JIOT.2019.2954738

[5] Li, C., Liu, Y., Xiao, J. & Zhou, J. Mceaaco-qsrp: A novel qos-secure routing protocol for industrial internet of things. *IEEE Internet Things J.* **9**(19), 18760–18777 (2022). 10.1109/JIOT.2022.3162106

[6] Jino Ramson, S. R. *et al.* A self-powered, real-time, lorawan iot-based soil health monitoring system. *IEEE Internet Things J.* **8**(11), 9278–9293 (2021). 10.1109/JIOT.2021.3056586

[7] Li, C. *et al.* A novel nature-inspired routing scheme for improving routing quality of service in power grid monitoring systems. *IEEE Syst. J.* **17**, 2616 (2022). 10.1109/JSYST.2022.3192856

[8] Gong, Y., Li, C., Wang, F. & Fang, X. Mhcf-cecso: A novel high-performance clustering framework for industrial iot. *IEEE Internet Things J.* **1**, 1 (2023).

[9] Al-Otaibi, S., Khan, R., Ali, J. & Ahmed, A. Artificial intelligence and internet of things-enabled decision support system for the prediction of bacterial stalk root disease in maize crop. *Comput. Intell.* **40**(1), e12632 (2024). 10.1111/coin.12632

[10] Jingjing Liu, X., Zhang, Z. L., Zhang, X., Jemric, T. & Wang, X. Quality monitoring and analysis of xinjiang ‘korla’ fragrant pear in cold chain logistics and home storage with multi-sensor technology. *Appl. Sci.* **9**(18), 3895 (2019). 10.3390/app9183895

[11] Sharma, H., Haque, A. & Jaffery, Z. A. Maximization of wireless sensor network lifetime using solar energy harvesting for smart agriculture monitoring. *Ad Hoc Netw.* **94**, 101966 (2019). 10.1016/j.adhoc.2019.101966

[12] Liu, Y. *et al.* Hpcp-qcwoa: High performance clustering protocol based on quantum clone whale optimization algorithm in integrated energy system. *Futur. Gener. Comput. Syst.* **135**, 315–332 (2022). 10.1016/j.future.2022.05.001

[13] Liu, Y. *et al.* Dcc-iacjs: A novel bio-inspired duty cycle-based clustering approach for energy-efficient wireless sensor networks. *J. King Saud Univ. Comput. Inf. Sci.* **35**(2), 775–790 (2023).

[14] Pundir, M. & Sandhu, J. K. A systematic review of quality of service in wireless sensor networks using machine learning: Recent trend and future vision. *J. Netw. Comput. Appl.* **188**, 103084 (2021). 10.1016/j.jnca.2021.103084

[15] Adil, M. *et al.* An efficient load balancing scheme of energy gauge nodes to maximize the lifespan of constraint oriented networks. *IEEE Access* **8**, 148510–148527 (2020). 10.1109/ACCESS.2020.3015941

[16] Liu, Y. *et al.* Qegwo: Energy-efficient clustering approach for industrial wireless sensor networks using quantum-related bioinspired optimization. *IEEE Internet Things J.* **9**(23), 23691–23704 (2022). 10.1109/JIOT.2022.3189807

[17] Luo, T. *et al.* An improved levy chaotic particle swarm optimization algorithm for energy-efficient cluster routing scheme in industrial wireless sensor networks. *Expert Syst. Appl.* **1**, 122780 (2023).

[18] Xu, M., Zu, Y., Zhou, J., Liu, Y. & Li, C. Energy-efficient secure qos routing algorithm based on elite niche clone evolutionary computing for wsn. *IEEE Internet Things J.* **1**, 1 (2024).

[19] Quy, N. M., Chehri, A., Quy, V. K. & Linh, D. M. A novel multi agents-based clustering algorithm for vanets in 5g networks. *Wirel. Netw.* **1**, 1–13 (2024).

[20] Zhiyi, Q. *et al.* An energy-efficient dynamic clustering protocol for event monitoring in large-scale wsn. *IEEE Sens. J.* **21**(20), 23614–23625 (2021). 10.1109/JSEN.2021.3103384

[21] García-Nájera, A., Zapotecas-Martínez, S. & Miranda, K. Analysis of the multi-objective cluster head selection problem in wsns. *Appl. Soft Comput.* **112**, 107853 (2021). 10.1016/j.asoc.2021.107853

[22] Sahoo, B. M., Pandey, H. M., Amgoth, T. & Gapso, H. A hybrid approach towards optimizing the cluster based routing in wireless sensor network. *Swarm Evol. Comput.* **60**, 100772 (2021). 10.1016/j.swevo.2020.100772

[23] Khan, A. I., Alsolami, F., Alqurashi, F., Abushark, Y. B. & Sarker, I. H. Novel energy management scheme in iot enabled smart irrigation system using optimized intelligence methods. *Eng. Appl. Artif. Intell.* **114**, 104996 (2022). 10.1016/j.engappai.2022.104996

[24] Ma, N., Zhang, H., Hang, H. & Qin, Y. Escvad: An energy-saving routing protocol based on voronoi adaptive clustering for wireless sensor networks. *IEEE Internet Things J.* **9**(11), 9071–9085 (2021). 10.1109/JIOT.2021.3120744

[25] Xiao, J., Li, C., Li, Z. & Zhou, J. Bs-scrm: A novel approach to secure wireless sensor networks via blockchain and swarm intelligence techniques. *Sci. Rep.* **14**(1), 9709 (2024). 38678073 10.1038/s41598-024-60338-6 PMC11589744

[26] Zheng, W.-M., Liu, N., Chai, Q.-W. & Liu, Y. Application of improved black hole algorithm in prolonging the lifetime of wireless sensor network. *Complex Intell. Syst.* **9**, 1–13 (2023). 10.1007/s40747-023-01041-3

[27] Zheng, W.-M., Lin-Dong, X., Pan, J.-S. & Chai, Q.-W. Cluster head selection strategy of wsn based on binary multi-objective adaptive fish migration optimization algorithm. *Appl. Soft Comput.* **148**, 110826 (2023). 10.1016/j.asoc.2023.110826

[28] Abdurohman, M., Supriadi, Y. & Fahmi, F. Z. A modified e-leach routing protocol for improving the lifetime of a wireless sensor network. *J. Inf. Process. Syst.* **16**(4), 845–858 (2020).

[29] Hassan, A.A.-H., Shah, W. M., Habeb, A.-H.H., Othman, M. F. I. & Al-Mhiqani, M. N. An improved energy-efficient clustering protocol to prolong the lifetime of the wsn-based iot. *Ieee Access* **8**, 200500–200517 (2020). 10.1109/ACCESS.2020.3035624

[30] Anitha, S. *et al.* Data transmission with improving lifetime of cluster network. *Turk. J. Comput. Math. Educ.* **12**(2), 420–428 (2021).

[31] Heidari, E., Movaghar, A., Motameni, H. & Barzegar, B. A novel approach for clustering and routing in wsn using genetic algorithm and equilibrium optimizer. *Int. J. Commun Syst* **35**(10), e5148 (2022). 10.1002/dac.5148

[32] Mehra, P. S., Doja, M. N. & Alam, B. Enhanced clustering algorithm based on fuzzy logic (e-cafl) for wsn: E-cafl for homogeneous wsn. *Scalable Comput. Pract. Exp.* **20**(1), 41–54 (2019). 10.12694/scpe.v20i1.1443

[33] Jin Wang, Yu., Gao, K. W., Sangaiah, A. K. & Lim, S.-J. An affinity propagation-based self-adaptive clustering method for wireless sensor networks. *Sensors* **19**(11), 2579 (2019). 31174313 10.3390/s19112579 PMC6603514

[34] Dattatraya, K. N., Raghava, K. & Rao, A. Hybrid based cluster head selection for maximizing network lifetime and energy efficiency in wsn. *J. King Saud Univ. Comput. Inf. Sci.* **34**, 716–726 (2022).

[35] Mittal, N., Singh, U., Salgotra, R. & Sohi, B. S. An energy efficient stable clustering approach using fuzzy extended grey wolf optimization algorithm for wsns. *Wirel. Netw.* **25**, 5151–5172 (2019). 10.1007/s11276-019-02123-2

[36] Cai, X., Geng, S., Di, W., Wang, L. & Qidi, W. A unified heuristic bat algorithm to optimize the leach protocol. *Concurr. Comput. Pract. Exp.* **32**(9), e5619 (2020). 10.1002/cpe.5619

[37] Mahajan, H. B. & Badarla, A. Cross-layer protocol for wsn-assisted iot smart farming applications using nature inspired algorithm. *Wirel. Pers. Commun.* **121**(4), 3125–3149 (2021). 10.1007/s11277-021-08866-6

[38] Sood, T. & Sharma, K. Luet: A novel lines-of-uniformity based clustering protocol for heterogeneous-wsn for multiple-applications. *J. King Saud Univ. Comput. Inf. Sci.* **34**(7), 4177–4190 (2022).

[39] Kotary, D. K., Nanda, S. J. & Gupta, R. A many-objective whale optimization algorithm to perform robust distributed clustering in wireless sensor network. *Appl. Soft Comput.* **110**, 107650 (2021). 10.1016/j.asoc.2021.107650

[40] Hemavathi, S. & Latha, B. Hflfo: Hybrid fuzzy levy flight optimization for improving qos in wireless sensor network. *Ad Hoc Netw.* **142**, 103110 (2023). 10.1016/j.adhoc.2023.103110

[41] Ali, H. *et al.* A novel metaheuristic for cluster head selection in wireless sensor networks. *IEEE Syst. J.* **15**(2), 2386–2397 (2020). 10.1109/JSYST.2020.2986811

